# Clinical and metabolomic predictors of regression to normoglycemia in a population at intermediate cardiometabolic risk

**DOI:** 10.1186/s12933-021-01246-1

**Published:** 2021-02-27

**Authors:** Magdalena del Rocío Sevilla-González, Jordi Merino, Hortensia Moreno-Macias, Rosalba Rojas-Martínez, Donají Verónica Gómez-Velasco, Alisa K. Manning

**Affiliations:** 1 Clinical and Translational Epidemiology Unit, Massachusetts General Hospital, 100 Cambridge, Boston, MA USA; 2 Programs in Metabolism and Medical & Population Genetics, Broad Institute of MIT and Harvard, Cambridge, MA USA; 3 Doctoral Program in Health Sciences, Universidad Nacional Autonóma de México, Mexico City, Mexico; 4 Department of Medicine, Harvard Medical School, Boston, MA USA; 5 Unidad de Investigacion en Enfermedades Metabolicas, Insituto Nacional de Ciencias Medicas y Nutricion “Salvador Zubiran”, Mexico City, Mexico; 6 Diabetes Unit and Center for Genomic Medicine, Massachusetts General Hospital, Boston, MA USA; 7 Universidad Autonoma Metropolitana, Mexico City, Mexico; 8 Insitituto Nacional de Salud Publica, Mexico City, Mexico

**Keywords:** Dysglycemia, Regression to normoglycemia, Metabolomics, Cardiometabolic risk

## Abstract

**Background:**

Impaired fasting glucose (IFG) is a prevalent and potentially reversible intermediate stage leading to type 2 diabetes that increases risk for cardiometabolic complications. The identification of clinical and molecular factors associated with the reversal, or regression, from IFG to a normoglycemia state would enable more efficient cardiovascular risk reduction strategies. The aim of this study was to identify clinical and biological predictors of regression to normoglycemia in a non-European population characterized by high rates of type 2 diabetes.

**Methods:**

We conducted a prospective, population-based study among 9637 Mexican individuals using clinical features and plasma metabolites. Among them, 491 subjects were classified as IFG, defined as fasting glucose between 100 and 125 mg/dL at baseline. Regression to normoglycemia was defined by fasting glucose less than 100 mg/dL in the follow-up visit. Plasma metabolites were profiled by Nuclear Magnetic Resonance. Multivariable cox regression models were used to examine the associations of clinical and metabolomic factors with regression to normoglycemia. We assessed the predictive capability of models that included clinical factors alone and models that included clinical factors and prioritized metabolites.

**Results:**

During a median follow-up period of 2.5 years, 22.6% of participants (n = 111) regressed to normoglycemia, and 29.5% progressed to type 2 diabetes (n = 145). The multivariate adjusted relative risk of regression to normoglycemia was 1.10 (95% confidence interval [CI] 1.25 to 1.32) per 10 years of age increase, 0.94 (95% CI 0.91–0.98) per 1 SD increase in BMI, and 0.91 (95% CI 0.88–0.95) per 1 SD increase in fasting glucose. A model including information from age, fasting glucose, and BMI showed a good prediction of regression to normoglycemia (AUC = 0.73 (95% CI 0.66–0.78). The improvement after adding information from prioritized metabolites (TG in large HDL, albumin, and citrate) was non-significant (AUC = 0.74 (95% CI 0.68–0.80), p value = 0.485).

**Conclusion:**

In individuals with IFG, information from three clinical variables easily obtained in the clinical setting showed a good prediction of regression to normoglycemia beyond metabolomic features. Our findings can serve to inform and design future cardiovascular prevention strategies.

**Supplementary Information:**

The online version contains supplementary material available at 10.1186/s12933-021-01246-1.

## Background

Impaired fasting glucose (IFG), a highly prevalent intermediate stage between normal glucose tolerance (NGT) and type 2 diabetes (T2D) [1], is characterized by metabolic alterations that lead to increased type 2 diabetes and cardiovascular complications [2, 3]. Empirical evidence support that individuals with type 2 diabetes are at twofold increased cardiovascular risk as compared to people without type 2 diabetes [4, 5], indication that preventing new onset of type 2 diabetes is an efficient approach to reduce the burden of cardiovascular disease. A number of studies have demonstrated the effectiveness of controlling cardiovascular risk factors in reducing the risk of cardiovascular outcomes among patients with diabetes and prediabetes [6–12]. The identification of clinical and molecular features associated with regression to normoglycemia has the potential to inform the design and implementation of more efficient cardiovascular risk-reduction strategies.

Preliminary evidence from prospective epidemiological studies have identified clinical predictors of regression to normoglycemia including age [13, 14], baseline fasting glucose [13–18], absence of postprandial hyperglycemia [13], higher insulin secretion [13, 17], lower BMI [17], preserved β-cell function [17, 18], lower fasting triglycerides [16, 17], and higher baseline muscle mass [16]. These studies have been mainly conducted in individuals from European or Asian ancestry, and the extent to which previous findings are similar in other populations with rapid conversion rate from impaired fasting glucose to type 2 diabetes such as Latino populations is unknown. The high prevalence of metabolic disorders in Amerindian-derived populations is a well-known phenomenon [19, 20]. Studies of household-level dietary patterns and eating habits in Mexico have confirmed an increased consumption of calories, fats and simple sugars [21]. Although these lifestyle changes have occurred in many areas of the world, Amerindian-derived populations exhibit a disproportionate impact on their overall health and rates of cardiometabolic diseases. The prevalence and incidence of type 2 diabetes among the Hispanic population in the United States are higher than the national average [22]. This has been attributed to the transition to a westernized lifestyle in which increased consumption of sugars and unhealthy fats maximizes inherited susceptibility obesity and insulin resistance [23].

In addition to clinical and genetic factors, recent evidence support the use of metabolomics to identify metabolic pathways or biomarkers of cardiometabolic risk. Recent studies have linked metabolites in specific metabolomic pathways to cardiometabolic risk [24, 25] and type 2 diabetes progression, for example: markers of amino acid catabolism [26–29], lipid oxidation [27, 29–32], or hexose metabolism [29, 33]. While the use of metabolomics has shed light on molecular mechanisms of increased cardiometabolic risk, the clinical use of metabolomics to identify individuals at increased cardiometabolic risk beyond conventional risk factors is still in its infancy. In two population-based studies, the inclusion of metabolites in a prediction model that included conventional risk factors barely improved the discriminative capability of the model with clinical variables [34, 35]. Findings were slightly different in a recent report in which the inclusion of metabolomic markers helped to identify additional people who might be at increased risk of type 2 diabetes [36]. Finally, the vast majority of these reports represent white American or European populations, and a recent report determined that metabolomic signatures associated with prediabetes may be distinct in African Americans (AA) and European Americans (EA) from that previously linked to type 2 diabetes in white individuals [37].

The role of circulating metabolites on the regression from impaired fasting glucose to a normoglycemic state is not fully understood. The use of these metabolites with clinical variables could provide for the identification of subjects who are more likely to regress from impaired fasting glucose to normoglycemia. Here, we analyzed longitudinal data of 9637 participants from the Mexican Study on Nutritional and Psychosocial Markers of Frailty [38] who were free of diabetes at baseline. We aimed to identify clinical and biological predictors of regression to normoglycemia and establish whether adding information from an NMR-based set of plasma metabolites could improve the predictive capability for regression to normoglycemia beyond clinical factors.

## Materials and methods

### Study design and populations

We used data from a prospective observational cohort study of Mexican adults living in large urban settings of central Mexico. The study sample was comprised of healthy adults ≥ 20 years old, with body mass index (BMI) ≥ 20 kg/m^2^, without previously diagnosed diabetes, cardiovascular disease, and cerebral vascular disease. Exclusion criteria included pregnancy or having an alcohol habit defined as consuming more than 10 servings of alcohol per week. Potential participants were evaluated at their workplaces (offices of the federal government or private companies), homes or during a visit to a relative in a medical unit. In the baseline visit, personal medical history, family history of type 2 diabetes, years of formal education, and socioeconomic status were recorded. The entire cohort was composed of 9637 participants with baseline evaluation. The follow-up examinations took place after a 3-year period (± 6 months). The response rate at follow-up was 63.7% (N = 6144). Impaired fasting glucose (IFG) and type 2 diabetes were defined according to the American Diabetes Association guidelines [39] using a measure of fasting plasma glucose between 100 and 125 mg/dL for prediabetes and ≥ 126 mg/dL for diabetes. The analysis sample was restricted to the IFG subset of the cohort.

The study was approved by the Ethics Committee of the Instituto Nacional de Ciencias Médicas y Nutrición Salvador Zubirán. Written informed consent was obtained from each participant. Our investigation and subsequent analyses were conducted in accordance with the Helsinki Declaration of Human Studies principles.

### Assessment of regression to normoglycemia

Regression to normoglycemia was defined as having one measurement of fasting glucose < 100 mg/dL at the follow-up visit and was assessed among individuals who were not taking glucose lowering medication including insulin. Incident type 2 diabetes was defined using the same criteria as baseline and expanded to include the World Health Organization criteria: having fasting plasma glucose ≥ 126 mg/dL, reporting taking any glucose lowering medication including insulin, or diagnosis of type 2 diabetes by a health professional.

### Assessment of clinical factors

Anthropometric measurements were conducted following standardized protocols. Subjects were evaluated while fasting, with light clothing and without shoes. Weight and height were used to compute BMI, as the ratio of weight (kilograms nearest 0.01) to squared height (m^2^). Waist and hip circumference (centimeters nearest 0.5) were measured at the midpoint between the lower ribs and the iliac crest, and at the level of the trochanter major respectively. Both were used to calculate the waist-hip index. Homeostasis Model Assessment for Insulin Resistance (HOMA-IR) was calculated using the following formula: fasting glucose (mg/dL) * fasting insulin/405) [40]. Homeostasis Model Assessment for cell-beta function (HOMA-B) was estimated with the following formula: 20 * fasting insulin (mmol/L) − 3.5 [40]. Insulin sensitivity was estimated also using METS-IR; it was computed as Ln((2 * fasting glucose (mg/dL)) + fasting triglycerides(mg/dL)) * body mass index (BMI))/(Ln(HDL-c)) [41]. Physical activity (physically active vs sedentary habit) was measured using the International Physical Activity Questionnaire [42]. Sedentary behavior is defined as any seated or reclined posture (e.g., sitting, lying down, and driving) that expends 1.50 or less Metabolic Equivalent Tasks (METs) while moderate to vigorous physical activity (MVPA) is any activity that expends 3.00 or more METs. Hypertension was defined as a systolic blood pressure (SBP) of ≥ 140 mmHg, or a diastolic blood pressure (DBP) of ≥ 90 mmHg, or taking antihypertensive medication, or self-report of previous diagnosis. Fasting triglycerides concentrations > 150 mg/dL were classified as hypertriglyceridemia. Obesity was defined BMI > 30 kg/m^2^, and abdominal obesity was classified according Adult Treatment Panel III [43], waist circumference > 102 cm in males and > 88 cm in females.

All serum samples kept frozen until processed in a central laboratory certified by the External Comparative Evaluation of Laboratories Program of the College of American Pathologists (Departamento de Endocrinología y Metabolismo, Instituto Nacional de Ciencias Médicas y Nutrición, México City). Blood samples were drawn from the radial vein after ~ 9 h fasting and were placed in EDTA-treated tubes (BD-vacutainer TM, London, UK). Samples were centrifuged for 15 min at 3000 rpm at 4 °C and stored at − 80 °C until the analysis. Serum concentration of glucose, total cholesterol, high-density lipoprotein cholesterol (HDL-c), were analyzed as was described previously [38].

### Metabolite determinations

Metabolites were analyzed by proton Nuclear Magnetic Resonance (NRM) in serum samples. The methodology has been previously described in detail [44]. In brief, the procedure defines three molecular windows to obtain information on (a) lipoprotein subclasses including chylomicrons or large VLDL particles), (b) serum lipid components including ω-3 and ω-6, poly, mono, and saturated fatty acids, phospholipids (PL), triglycerides (TG), cholesterol (C), free cholesterol (FC), and cholesterol esters (CE), apolipoprotein A-I and APOB, and (c) low molecular weight components including alanine, glutamine, glycine histidine, isoleucine, leucine, valine, phenylalanine, tyrosine, acetate, acetoacetate, 3-hydroxybutyrate, creatinine, albumin and glycoprotein acetyl (a-1 acid glycoprotein). Quality control procedures were performed according a previous metabolomics pipeline including the following steps: (1) removing metabolites with > 25% missing data, (2) log-transforming the remaining metabolites, and (3) performing rank-based inverse normal transformation to calculate metabolite abundance z-scores.

### Statistical analysis

The study sample was categorized into three groups according to glycemic status at the end of follow-up: regression to normoglycemia, impaired fasting glucose maintenance, or progression to type 2 diabetes. Comparisons between these three groups were tested with one-way ANOVA for continuous variables or with chi-square test for qualitative variables. Clinical categorical variables were reported as frequencies and percentages. Quantitative variables were reported as means and standard deviation for normal distributed variables or median and interquartile range for non-normal distributed variables.

We used multivariable Cox proportional hazards models to calculate hazard ratios (HR), and 95% confidence interval (95% CI) for regression to normoglycemia for clinical and metabolomic factors. Variables with evidence of nominal significant association in the bivariate analysis, that did not exhibit collinearity (variance inflation factor > 7), were selected for inclusion in predictive models. The best model was selected as the set of variables with the highest discriminatory capability according to the C statistic and the area under the receiver operating characteristic curve (ROC). To correct overfitting in the AUC, we used 2000 stratified bootstrap replicated to generate 95% confidence intervals. We tested the difference between the predictive capability of estimates from the clinical and clinical + metabolomic models with the DeLong test [45]. Sensitivity analyses were conducted to investigate associations with HOMA-IR and HOMA-B using linear regression modeling accounting for follow-up time, confounders, and corresponding baseline values. We considered two sided α level of 0.05 for all analyses. All analyses were conducted using R software version 3.4.3.

## Results

From 9637 participants included in the primary cohort, a total of 491 individuals were eligible for this analysis based on their impaired fasting glucose status at baseline. The most frequent cause of exclusion from the primary cohort was glycemia < 100 mg/dL at baseline, which excluded 56.43% participants (N = 2808). After ~ 2.5 years of follow-up, n = 111 (22.6% of N = 491) participants regressed to normoglycemia, while 235 (47.9%) remained as impaired fasting glucose and 145 (29.5%) progressed to type 2 diabetes. Baseline characteristics from included participants according to glycemic phenotypes at the end of follow-up are shown in Table 1. Compared with individuals who progressed to type 2 diabetes or maintained their impaired fasting glucose status, individuals who regressed to normoglycemia had lower fasting glucose concentrations (p < 0.001), and lower BMI (p = 0.049).

**Table 1:** Baseline characteristics between groups according final glucose status (n = 491)

	Overall (n = 491)	Regression to normoglycemia (n = 111)	IFG maintenance (n = 235)	Progressed to T2D (n = 145)	*p* value
Age (years)	48.72 ± 11.02	49.98 ± 11.2	48.35 ± 11.00	48.34 ± 10.87	0.27
Women n, (%)	327, (66.59)	75, (67.5)	161, (68.5)	91 (62.7)	0.498
Hypertension	341 (69.45)	76, (68.4)	173, (73.6)	92, (63.4)	0.262
Years of education	11.72 ± 5.01	12.52 ± 5.41	11.61 ± 4.99	11.29 ± 4.68	0.061
BMI (kg/m^2^)	30.15 [6.3]	29.45[6.4]	30.00 [5.5]	30.78 [7.25]	0.049*
Obesity n, (%)	248 (50.5)	51 (45.9)	116 (49.36)	81 (55.86)	0.258
Abdominal obesity n, (%)	298 (60.6)	58 (52.2)	144 (61.27)	96 (66.2)	0.074
Sedentary habit n, (%)	348 (70.8)	76, (68.4)	169, (72)	103, (71)	0.804
Fasting glucose (mg/dL)	105.00 [8]	103.00 [6]	105.00 [8]	108.00 [12]	0.001*
Fasting insulin (UI)	14.80 [11.2]	13.80 [11.05]	15.05 [10.2]	14.6 [11.5]	0.508
HOMA-IR	3.92 [2.9]	3.6 [2.9]	4.0 [2.7]	3.95 [3.28]	0.285
HOMA-B	46.83 [37.23]	44.50 [39.91]	47.41 [34.6]	46.02 [38.67]	0.673
METS-IR	49.40 [12.11]	48.45 [13]	49.44 [11.3]	50.48 [12.2]	0.289
Triglycerides (mg/dL)	197.00 [121]	200.00 [169]	188.00 [112]	205.00 [116]	0.542
Hypertriglyceridemia n, (%)	355 (72.3)	76 (68.4)	169 (71.9)	110 (75.86)	0.417
Total-cholesterol (mg/dL)	217.42 ± 42.70	224.69 ± 45.3	214.39 ± 43	216.76 + 39.44	0.184
HDL-c (mg/dL)	42.14 ± 10.65	43.05 ± 10.11	41.29 ± 10.10	42.82 ± 11.84	0.987
LDL-c (mg/dL)	134.51 ± 32.74	138.78 ± 31.0	133.77 ± 35.04	132.60 + 29.83	0.232
Apolipoprotein-B (mg/dL)	116.00 [38.4]	120.0 [36.5]	115.00 [39.35]	115.00 [37]	0.712

Abdominal obesity male > 102 cm, female > 88 cm. Hypertension: > 130 mmHg or > 88 mmHg or taking antihypertensive medication, or self-report of previous diagnosis

*ATP III* adult treatment panel III, *IFG* impaired fasting glucose IFG, *T2D* type 2 diabetes, *BMI* body mass index (kilogram/height^2^), *HDL-C* high-density lipoprotein cholesterol, *LDL-c* low-density lipoprotein-cholesterol,* HOMA-IR* Homeostasis Model Assessment for Insulin Resistance (fasting glucose * fasting insulin/405), hypertriglyceridemia: fasting triglycerides > 150 mg/dL

One-way ANOVA was used to compute the *p value* between three groups

### Clinical variables associated with regression to normoglycemia

The multivariable-adjusted hazard ratio (HR) of regression to normoglycemia was 1.10 (95% confidence interval [CI] 1–1.03) per 10 years of age, 0.94 (95% CI 0.91–0.98) per 1 standard deviation (SD) increase in BMI, 0.91 (95% CI 0.88–0.95) per 1 SD increase in fasting glucose, 0.91 (95% CI 0.84 to 0.99) per 1SD increase in HOMA-IR, and 0.99 (95% CI 0.94–0.98) per 1 SD increase in METS-IR (Table 2).

**Table 2:** Cox proportional hazard model regression results of clinical factors associated with regression to normoglycemia

	HR	CI 95%	*p* value
Age (10 years)	1.019	1.10–1.30	0.044
Sex	0.779	0.52–1.16	0.226
Hypertension	0.819	0.54–1.23	0.341
Years of education	0.994	0.95–1.03	0.771
BMI (kg/m^2^)	0.947	0.91–0.98	0.009
Obesity n, (%)	0.630	0.43–0.92	0.017
Abdominal obesity n, (%)	0.586	0.40–0.85	0.006
Sedentary habit n, (%)	0.768	0.51–1.15	0.206
Fasting glucose (mg/dL)	0.919	0.883–0.956	0.001
Fasting insulin (UI)	0.980	0.95–1.002	0.070
HOMA-IR	0.916	0.845–0.99	0.03
HOMA-B	0.995	0.98–1.02	0.139
METS-IR	0.996	0.94–0.98	0.002
Triglycerides (mg/dL)	0.99	0.99 – 1.00	0.863
Hypertriglyceridemia n, (%)	0.75	0.50–1.12	0.168
Total-cholesterol (mg/dL)	1.002	0.99–1.00	0.259
HDL-c (mg/dL)	1.01	0.99–1.02	0.169
LDL-c (mg/dL)	1.04	0.99–1.01	0.271
Apolipoprotein-B (mg/dL)	1.01	0.99- 1.08	0.742

*BMI* body mass index

*p* value was computed in a Cox regression model comparing subjects who regress to normoglycemia and subjects to progressed to type 2 diabetes (T2D)

### Metabolomic variables associated with regression to normoglycemia

**Fig. 1:**
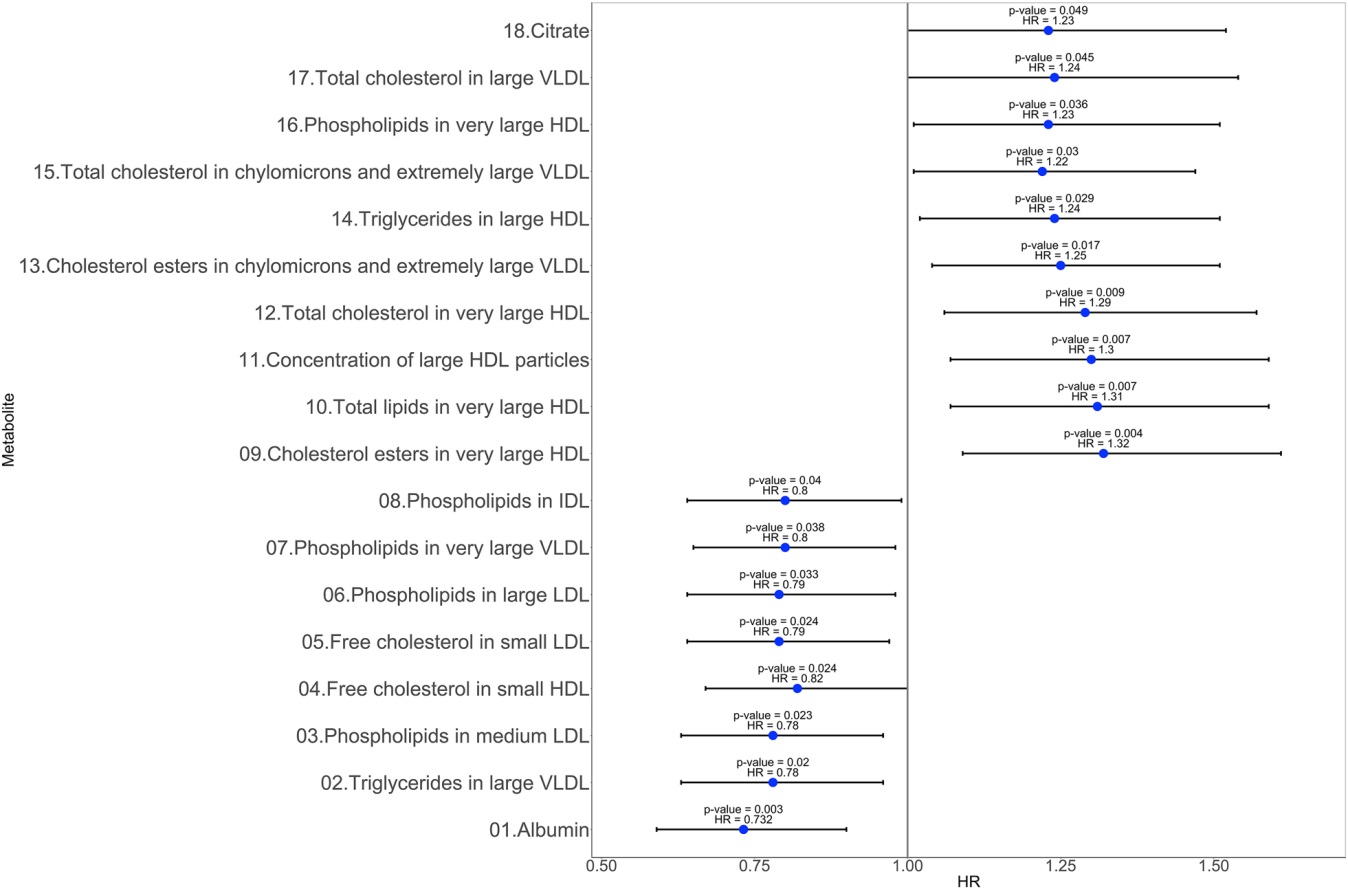
Metabolites associated with regression to normoglycemia (p < 0.05). HR: hazard ratio and their CI 95%, models were adjusted by baseline variables: age, sex, body mass index, and fasting glucose

**Fig. 2:**
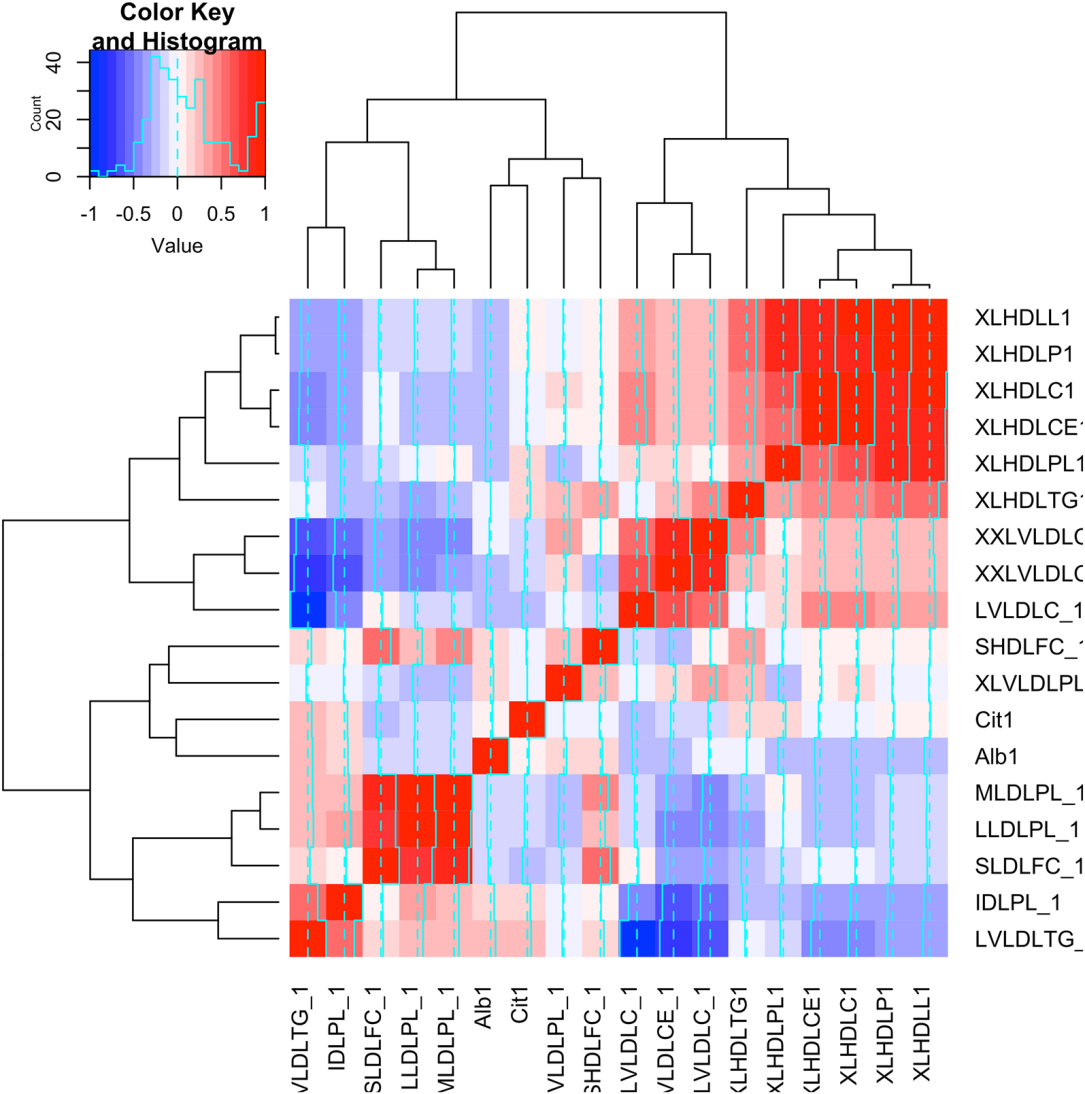
Correlation matrix with eighteen metabolites associated with normal glucose tolerance regression

After adjustment for potential confounders, we identified 18 metabolites associated with regression to normoglycemia in models adjusting for baseline age, sex, BMI, and fasting glucose (Fig. 1). Ten metabolites showed a positive association with regression to normoglycemia, most of them capturing different HDL composition characteristics (hazard ratios [HR] ranging from 1.22 to 1.32 per 1 SD increment). Eight metabolites showed a negative association with regression to normoglycemia, including three LDL composition-related and two VLDL composition-related (HR ranging from 0.72 to 0.80 per 1 SD increment; Fig. 2).

The multivariable adjusted relative risk of regression to normoglycemia was 1.32 (95% CI 1.09–1.61) per 1 SD-increment in cholesterol esters in very large HDL, 1.31 (95% CI 1.07–1.59) per 1 SD-increment in total lipids in very large HDL, 1.30, (95% CI 1.07–1.59) per 1 SD-increment concentration of large HDL particles, 1.29 (95% CI 1.06–1.57) per 1 SD-increment in total cholesterol in very large HDL, and 0.73 (95% CI 0.59–0.90) per 1 SD-increment in albumin, In addition, we showed that some metabolites featuring LDLc composition lipoproteins were inversely associated with regression to normoglycemia including phospholipids in medium LDL, 0.78 (95% CI 0.63–0.96), free cholesterol in small LDL 0.79 (95% CI 0.64–0.98), phospholipids in large LDL, 0.79 (95% CI 0.64–0.98). In a sensitive analysis, we identified twenty-five metabolites associated with HOMA-IR and/or HOMA-B levels (Table 3).

**Table 3:** Metabolites at baseline associated with HOMA-IR and HOMA-B at follow-up

Metabolites	Metabolites associated with HOMA-IR				Metabolites associated with HOMA-B			
	β	SE	CI 95%	*p* value	β	SE	CI 95%	*p* value
*Phospholipids in large HDL*	− 0.14	0.04	− 0.24 to − 0.04	0.003	− 0.11	0.04	− 0.20 to − 0.02	0.011
*Concentration of large HDL particles*	− 0.15	0.04	− 0.24 to − 0.05	0.002	− 0.11	0.04	− 0.20 to − 0.02	0.012
*Total lipids in very large HDL*	− 0.15	0.04	− 0.24 to − 0.05	0.002	− 0.12	0.10	− 0.20 to − 0.02	0.008
Creatinine	0.10	0.04	0.005 to 0.20	0.038	0.12	0.04	0.03 to 0.21	0.009
Mean diameter for HDL particles	− 0.12	0.04	− 0.22 to − 0.03	0.007	− 0.10	0.04	− 0.19 to − 0.01	0.021
Free cholesterol in large HDL	− 0.13	0.05	− 0.23 to − 0.03	0.008	− 0.11	0.04	− 0.21 to − 0.02	0.012
*Cholesterol esters in very large HDL*	0.09	0.04	0.001 to 0.19	0.04				
Free cholesterol in very large HDL	− 0.15	0.04	− 0.24 to − 0.05	0.001	− 0.11	0.04	− 0.20 to − 0.02	0.012
Total cholesterol in large HDL	− 0.13	0.05	− 0.23 to − 0.03	0.008	− 0.12	0.04	− 0.21 to − 0.09	0.010
Cholesterol esters in large HDL	− 0.13	0.05	− 0.23 to − 0.03	0.008	− 0.12	0.04	− 0.21 to − 0.03	
Total lipids in large HDL	− 0.12	0.05	− 0.22 to − 0.03	0.010	− 0.12	0.04	− 0.21 to − 0.02	0.010
Concentration of large HDL particles	− 0.12	0.05	− 0.22 to − 0.03	0.010	− 0.12	0.04	− 0.21 to − 0.02	0.010
Phospholipids in medium HDL	− 0.13	0.05	− 0.22 to − 0.03	0.009	− 0.12	0.04	− 0.21 to − 0.03	0.009
*Total cholesterol in very large HDL*	− 0.12	0.04	− 0.21 to − 0.02	0.014	–	–		–
Total cholesterol in HDL2	− 0.11	0.04	− 0.21 to − 0.02	0.016	− 0.09	0.04	− 0.18 to − 0.009	0.03
Total cholesterol in HDL	− 0.10	0.04	− 0.20 to − 0.01	0.02	− 0.09	0.04	− 0.18 to − 0.002	0.04
Free cholesterol in medium HDL	− 0.10	0.04	− 0.20 to − 0.009	0.03	–	–		–
Cholesterol esters in very large HDL	− 0.10	0.04	− 0.20 to − 0.009	0.03	–	–		–
Mean diameter for LDL particles	0.10	0.04	0.009 to 0.20	0.03	0.010	0.04	0.01 to 0.19	0.021
Free cholesterol in very large HDL	− 0.10	0.04	− 0.19 to − 0.005	0.03	–	–		–
Cholesterol esters in small VLDL	0.10	0.04	0.005 to 0.20	0.03	–	–		–
Total cholesterol in small VLDL	0.10	0.04	0.004 to 0.20	0.04	–	–		–
Triglycerides in medium HDL	0.10	0.04	0.002 to 0.19	0.04				
Triglycerides in small VLDL					− 0.09	0.04	− 0.18 to − 0.001	0.04
Triglycerides in medium HDL					− 0.10	0.04	− 0.19 to − 0.01	0.02

Italic metabolites represent those associated with normal fasting glucose regression

*CI* 95% interval confidence, *SE* standard error

p values were computed with a lineal regression model considering as confounders: age, sex, body mass index, and baseline values: HOMA-IR and HOMA-B respectively

### Predictors of regression to normoglycemia

**Fig. 3:**
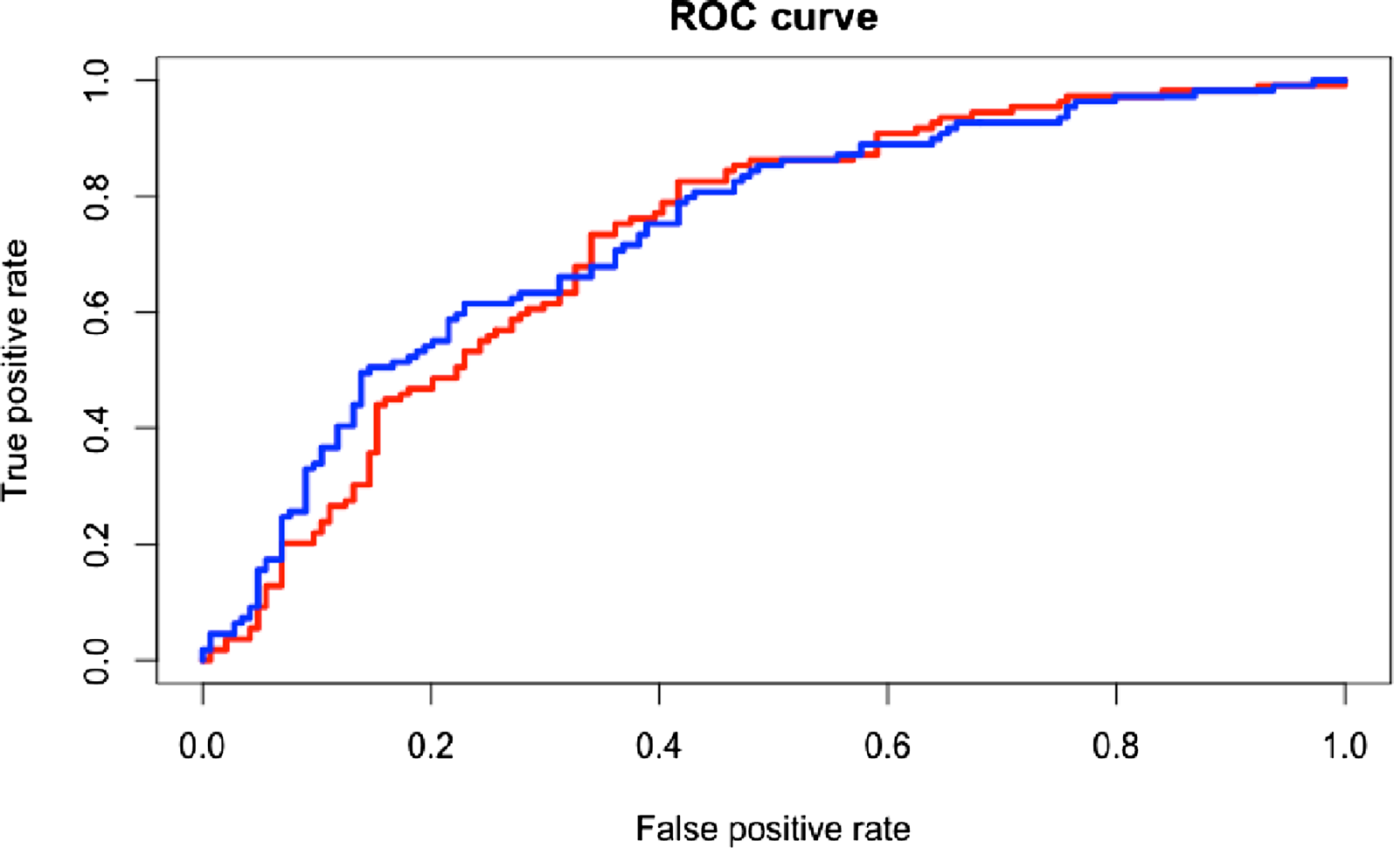
Area under the ROC curve predicting the regression to normoglycemia. Red line represents the predictive capability of clinical variables (age, body mass index and fasting glucose) for which the AUC was 0.73 (95% CI 0.66–0.78). Blue line represents the model with clinical and metabolomic variables: albumin, citrate, and triglycerides in large HDL 0.74 (95% CI 0.68–0.80, p = 0.485)

The clinical variables with higher performance to predict regression to normoglycemia were age, fasting glucose, and BMI (AUC = 0.727, 95% CI 0.66–0.78). We next investigated whether adding uncorrelated metabolomic features to the clinical prediction model improved the predictive capability of the model. We showed that adding information from triglycerides in large HDL, albumin, citrate, increased the AUC to 0.744 but the difference with the clinical prediction model was not significant (95% CI 0.68–0.80; p = 0.485; Fig. 3).

## Discussion

Here, we inform the regression and progression rates of dysglycemia in a set of adults with impaired fasting glucose living in urban centers of Central Mexico. After ~ 2.5 years of follow-up, 22.6% subjects regressed to normoglycemia, 36% remained as impaired fasting glucose and 22.9% progressed to type 2 diabetes. We showed that age, lower BMI, and lower glycemia were the main clinical predictors associated with regression to normoglycemia, and that the addition of NMR-based set of metabolomics biomarkers, did not significantly improved the predictive capability over the model that included clinical variables alone. Taken together, our findings may have implications for cardiovascular prevention strategies as they identify a set of clinical features that are associated with less likelihood for developing type 2 diabetes among individuals with impaired fasting glucose.

### Rates of regression to normoglycemia

To our knowledge, this is the first report of the rate of regression in Latin-American populations, a population that is characterized by the high conversation rates from impaired fasting glucose to type 2 diabetes. There are few studies that have focused on regression to normoglycemia despite it is the most common and profitable outcome in the midterm and long-term respectively. Our results allow us to compare the regression rates in Mexicans compared against other populations. The rate found in this study is higher to what was found in an Asian and in a multiethnic cohort at 1 and 10 years of follow-up [15, 46]. Our findings concurs with data from the Diabetes Prevention Program (DPP) showing that within treatment groups, normoglycemia was attained once in 23% (170/736), 25% (161/647) and 23% (137/607), in intensive life style, metformin, and placebo treatment arms, respectively [17]. Our findings reinforce the identification of individuals with impaired fasting glucose to advance the prevention of type 2 diabetes, to overcome the burden of cardiometabolic complications. The identification of the variables that predict a higher likelihood for having regression may be useful to prioritize access to care, particularly in populations where the medical access is limited. Our findings confirm the importance of fasting glucose and BMI in the profile of the subjects who achieve regression to normoglycemia and expand them to age, which has been shown very relevant for maintaining normal glucose levels [47, 48].

### Metabolomics and metabolic dysfunction

A novel contribution of our study is that we investigated the associations of circulating metabolites with regression to normoglycemia, in a Hispanic population (Additional file 1: Table S1). In this study we identified 18 associated with regression to normoglycemia. Metabolites associated with regression to normoglycemia highlight features of lipid components. Several studies have found evidence of changes in lipid coregulation existing before diabetes onset [27, 49], principally with triacylglycerols (TAGs), lyso-phosphatidylinositols, phosphatidylcholines. In this study, we showed that phospholipids in medium or large LDL particles of free cholesterol in small LDL were associated with lower likelihood to regress to normoglycemia, while lipid components in large HDLc particles were associated with increased likelihood to regress to normoglycemia. Although, there is lack of information regarding the clinical implications and usability of HDL particles, some evidence describes a negative relationship between the number of large HDL particles and cardiovascular disease, and conversely, a reduced mean HDL size is equally associated with cardiovascular disease in large-scale clinical studies [50]. This direction of the effect was confirmed by our study in the associations with insulin sensitivity and secretion where larger HDL particles had a negative association with insulin sensitivity in subject who remained as impaired fasting glucose or regressed to normoglycemia. Our findings support evidence of the clinical usability of a detailed lipid metabolomic profiling and their clinical consequences. Although further studies are needed, HDL composition profile can be used as biomarkers of cardiovascular deterioration even in an early state such as impaired fasting glucose.

In our study, we found positive associations of several lipoprotein metabolites with regression to normoglycemia: total cholesterol in large VLDL, total cholesterol in chylomicrons and extremely large VLDL, cholesteryl esters in chylomicrons, and extremely large VLDL. Previous findings suggest that the genetic underpinnings of mean lipoprotein diameter differ by race/ethnicity. SNPs in APOB gene region had been significantly associated with mean VLDL diameter in Hispanics, our VLDL results might be a footprint of these associations [51].

In this report, the prioritized NMR metabolites did not improve the predictive capability to regress to normoglycemia. These findings may be somewhat limited by the coverage of plasma metabolites and lipids available in our metabolomics platform. Recent data suggest that more granular lipid panels including triglycerides or phospholipids with different acyl chain lengths and saturation are differently associated with T2D risk [27, 49], hence our findings need to be interpreted with caution.

In our study we found that individuals with low concentrations of albumin at baseline were more likely to regress to normoglycemia, even adjusting for age, BMI and fasting glucose concentrations (p < 0.003). Serum albumin is the main protein of the plasma, its main function is the regulation of the colloidal osmotic pressure of the blood [52]. Previous reports show inverse associations with type 2 diabetes-related traits [53, 54]. Some differences might lie in the studied sample size, most of studies have been studied the risk of healthy individuals to develop type 2 diabetes, whereas our sample is composed by high-risk subjects with impaired fasting glucose. Therefore, the protective capability conferred by the albumin thought its role as antioxidant might be diminished.

### Limitations and future directions

There are several limitations in our study including the use of only one measurement of fasting glucose measurement to define regression to normoglycemia. This increases the likelihood to fall under the regression toward the mean bias [55]. Another limitation of this study is the use of a metabolomics panel that included only a limited number of metabolites. The inclusion of other metabolomic species such as the ones available in a non-targeted metabolomics platforms or the implementation of a broader lipid panel could yield different results. One of the main limitations of this study is that dietary information was not available. Although it is possible that residual confounding by dietary modifications might exist in our study, participants were not instructed to follow any specific diet. Our findings need to be confirmed in independent studies and other populations at high risk of type 2 diabetes, with more granular data and extended follow-up. Finally, additional studies to test the treatment response such as diet and physical activity will complement the evidence in regards the usability of these variables.

## Conclusions

The findings from this study provide quantitative evidence on the progression and regression rates of dysglycemia and identify a set of clinical features that are associated with regression to normoglycemia among individuals with impaired fasting glucose. We also provide evidence about the role of specific lipoproteins subtypes on the regression to normoglycemia and highlight the role of HDL and LDL particles composition. Yet, we showed that the addition of an NMR-based set of metabolomics information did not improve the capability to predict regression to normoglycemia. Our findings can serve to inform and design future strategies to advance the prevention to type 2 diabetes and related cardiometabolic complications.

## Supplementary Information

**Additional file 1: Table S1.** Metabolites associated with regression to normoglycemia compared with progression to type 2 diabetes.

## Data Availability

The datasets used and/or analyzed during the current study are available from the corresponding author on reasonable request.

## Abbreviations

BMI: Body mass index
IFG: Impaired fasting glucose
HDL: High density lipoprotein
LDL: Low density lipoprotein
VLDL: Very low-density lipoprotein
METs: Metabolic Equivalent Tasks
HOMA: Homeostasis model assessment
NMR: Nuclear magnetic resonance
AUC: Area under the curve
HR: Hazard ratio

## References

[1] Cho NH, Shaw JE, Karuranga S, Huang Y, da Rocha Fernandes JD, Ohlrogge AW (2018). IDF diabetes atlas: global estimates of diabetes prevalence for 2017 and projections for 2045. Diabetes Res Clin Pract.

[2] Abdul-Ghani M, DeFronzo RA, Jayyousi A (2016). Prediabetes and risk of diabetes and associated complications. Curr Opin Clin Nutr Metab Care.

[3] Huang Y, Cai X, Mai W, Li M, Hu Y (2016). Association between prediabetes and risk of cardiovascular disease and all cause mortality: systematic review and meta-analysis. BMJ.

[4] Haffner SM, Lehto S, Rönnemaa T, Pyörälä K, Laakso M (1998). Mortality from coronary heart disease in subjects with type 2 diabetes and in nondiabetic subjects with and without prior myocardial infarction. N Engl J Med.

[5] Sarwar N, Gao P, Seshasai SRK, Gobin R, Kaptoge S, Di Angelantonio E (2010). Diabetes mellitus, fasting blood glucose concentration, and risk of vascular disease: a collaborative meta-analysis of 102 prospective studies. Lancet.

[6] Turner R (1998). Effect of intensive blood-glucose control with metformin on complications in overweight patients with type 2 diabetes (UKPDS 34). Lancet.

[7] Gæde P, Vedel P, Larsen N, Jensen GVH, Parving H-H, Pedersen O (2003). Multifactorial intervention and cardiovascular disease in patients with type 2 diabetes. N Engl J Med.

[8] Colhoun HM, Betteridge DJ, Durrington PN, Hitman GA, Neil HAW, Livingstone SJ (2004). Primary prevention of cardiovascular disease with atorvastatin in type 2 diabetes in the collaborative atorvastatin diabetes study (CARDS): multicentre randomised placebo-controlled trial. Lancet.

[9] Holman RR, Paul SK, Bethel MA, Neil HAW, Matthews DR (2008). Long-term follow-up after tight control of blood pressure in type 2 diabetes. N Engl J Med.

[10] Fulcher J, O’Connell R, Voysey M, Emberson J, Blackwell L, Mihaylova B (2015). Efficacy and safety of LDL-lowering therapy among men and women: meta-analysis of individual data from 174 000 participants in 27 randomised trials. Lancet.

[11] Merino J, Leong A, Posner DC, Porneala B, Masana L, Dupuis J (2017). Genetically driven hyperglycemia increases risk of coronary artery disease separately from type 2 diabetes. Diabetes care.

[12] Leong A, Chen J, Wheeler E, Hivert MF, Liu CT, Merino J (2019). Mendelian randomization analysis of hemoglobin A1c as a risk factor for coronary artery disease. Diabetes care.

[13] Perreault L, Kahn SE, Christophi CA, Knowler WC, Hamman RF, Diabetes Prevention Program Research Group (2009). Regression from pre-diabetes to normal glucose regulation in the diabetes prevention program. Diabetes Care.

[14] Herman WH, Pan Q, Edelstein SL, Mather KJ, Perreault L, Barrett-Connor E (2017). Impact of lifestyle and metformin interventions on the risk of progression to diabetes and regression to normal glucose regulation in overweight or obese people with impaired glucose regulation. Diabetes Care.

[15] Bodicoat DH, Khunti K, Srinivasan BT, Mostafa S, Gray LJ, Davies MJ (2017). Incident Type 2 diabetes and the effect of early regression to normoglycaemia in a population with impaired glucose regulation. Diabet Med.

[16] Hwang Y-C, Cho I-J, Jeong I-K, Ahn KJ, Chung HY (2018). Factors associated with regression from prediabetes to normal glucose tolerance in a Korean general population: a community-based 10-year prospective cohort study. Diabet Med.

[17] Perreault L, Pan Q, Mather KJ, Watson KE, Hamman RF, Kahn SE (2012). Effect of regression from prediabetes to normal glucose regulation on long-term reduction in diabetes risk: results from the diabetes prevention program outcomes study. Lancet.

[18] Nanditha A, Ram J, Snehalatha C, Selvam S, Priscilla S, Shetty AS (2014). Early improvement predicts reduced risk of incident diabetes and improved cardiovascular risk in prediabetic Asian Indian men participating in a 2-year lifestyle intervention program. Diabetes Care.

[19] Aguayo-Mazzucato C, Diaque P, Hernandez S, Rosas S, Kostic A, Caballero AE (2019). Understanding the growing epidemic of type 2 diabetes in the Hispanic population living in the United States. Diabetes Metab Res Rev.

[20] Aguilar-Salinas CA, Tusie-Luna T, Pajukanta P (2014). Genetic and environmental determinants of the susceptibility of Amerindian derived populations for having hypertriglyceridemia. Metabolism.

[21] Flores M, Macias N, Rivera M, Lozada A, Barquera S, Rivera-Dommarco J (2010). Dietary patterns in Mexican adults are associated with risk of being overweight or obese. J Nutr.

[22] Yang W, Dall TM, Beronjia K, Lin J, Semilla AP, Chakrabarti R (2018). Economic costs of diabetes in the US in 2017. Diabetes Care.

[23] Williams AL, Jacobs SBR, Moreno-Macías H, Huerta-Chagoya A, Churchhouse C, Consortium TST 2 D (2013). Sequence variants in SLC16A11 are a common risk factor for type 2 diabetes in Mexico. Nature.

[24] Katakami N, Katakami N, Omori K, Taya N, Arakawa S, Takahara M (2020). Plasma metabolites associated with arterial stiffness in patients with type 2 diabetes. Cardiovasc Diabetol.

[25] Uddin GM, Zhang L, Shah S, Fukushima A, Wagg CS, Gopal K (2019). Impaired branched chain amino acid oxidation contributes to cardiac insulin resistance in heart failure. Cardiovasc Diabetol.

[26] Wang TJ, Larson MG, Vasan RS, Cheng S, Rhee EP, McCabe E (2011). Metabolite profiles and the risk of developing diabetes. Nat Med.

[27] Rhee EP, Cheng S, Larson MG, Walford GA, Lewis GD, McCabe E (2011). Lipid profiling identifies a triacylglycerol signature of insulin resistance and improves diabetes prediction in humans. J Clin Invest.

[28] Wang TJ, Ngo D, Psychogios N, Dejam A, Larson MG, Vasan RS (2013). 2-Aminoadipic acid is a biomarker for diabetes risk. J Clin Invest.

[29] Floegel A, Stefan N, Yu Z, Mühlenbruch K, Drogan D, Joost H-G (2013). Identification of serum metabolites associated with risk of type 2 diabetes using a targeted metabolomic approach. Diabetes.

[30] Newgard CB (2012). Interplay between lipids and branched-chain amino acids in development of insulin resistance. Cell Metab.

[31] Zeng Y, Mtintsilana A, Goedecke JH, Micklesfield LK, Olsson T, Chorell E (2019). Alterations in the metabolism of phospholipids, bile acids and branched-chain amino acids predicts development of type 2 diabetes in black South African women: a prospective cohort study. Metabolism.

[32] Godzien J, Kalaska B, Adamska-Patruno E, Siroka J, Ciborowski M, Kretowski A (2019). Oxidized glycerophosphatidylcholines in diabetes through non-targeted metabolomics: their annotation and biological meaning. J Chromatogr B Anal Technol Biomed Life Sci.

[33] Drogan D, Dunn WB, Lin W, Buijsse B, Schulze MB, Langenberg C (2015). Untargeted metabolic profiling identifies altered serum metabolites of type 2 diabetes mellitus in a prospective, nested case control study. Clin Chem.

[34] Walford GA, Porneala BC, Dauriz M, Vassy JL, Cheng S, Rhee EP (2014). Metabolite traits and genetic risk provide complementary information for the prediction of future type 2 diabetes. Diabetes Care.

[35] Vangipurapu J, Fernandes Silva L, Kuulasmaa T, Smith U, Laakso M (2020). Microbiota-related metabolites and the risk of type 2 diabetes. Diabetes Care.

[36] Khan SR, Manialawy Y, Obersterescu A, Cox BJ, Gunderson EP, Wheeler MB (2020). Diminished sphingolipid metabolism, a hallmark of future type 2 diabetes pathogenesis, is linked to pancreatic b cell dysfunction pancreatic beta-cell dysfunction glucose insulin release cell death prognostic biomarker for T2D T2D development. IScience.

[37] Owei I, Umekwe N, Stentz F, Wan J, Dagogo-Jack S (2019). Amino acid signature predictive of incident prediabetes: a case-control study nested within the longitudinal pathobiology of prediabetes in a biracial cohort. Metabolism.

[38] Ruiz-Arregui L, Ávila-Funes JA, Amieva H, Borges-Yáñez SA, Villa-Romero A, Aguilar-Navarro S (2013). The Coyoacán cohort study: design, methodology, and participants’ characteristics of a mexican study on nutritional and psychosocial markers of frailty. J Frailty Aging.

[39] Association AD (2010). Standards of medical care in diabetes-2010. Diabetes care.

[40] Matthews DR, Hosker JP, Rudenski AS, Naylor BA, Treacher DF, Turner RC (1985). Homeostasis model assessment: insulin resistance and beta-cell function from fasting plasma glucose and insulin concentrations in man. Diabetologia.

[41] Bello-Chavolla OY, Almeda-Valdes P, Gomez-Velasco D, Viveros-Ruiz T, Cruz-Bautista I, Romo-Romo A (2018). METS-IR, a novel score to evaluate insulin sensitivity, is predictive of visceral adiposity and incident type 2 diabetes. Eur J Endocrinol.

[42] Craig CL, Marshall AL, Sjöström M, Bauman AE, Booth ML, Ainsworth BE (2003). International physical activity questionnaire: 12-country reliability and validity. Med Sci Sport Exerc.

[43] Huang PL (2009). A comprehensive definition for metabolic syndrome. Dis Model Mech.

[44] Inouye M, Kettunen J, Soininen P, Silander K, Ripatti S, Kumpula LS (2010). Metabonomic, transcriptomic, and genomic variation of a population cohort. Mol Syst Biol.

[45] DeLong ER, DeLong DM, Clarke-Pearson DL (1988). Comparing the areas under two or more correlated receiver operating characteristic curves: a nonparametric approach. Biometrics.

[46] Guo VY, Yu EY, Wong CK, Sit RW, Wang JH, Ho SY (2016). Validation of a nomogram for predicting regression from impaired fasting glucose to normoglycaemia to facilitate clinical decision making. Fam Pract.

[47] Lindstrom J, Louheranta A, Mannelin M, Rastas M, Salminen V, Eriksson J (2003). The Finnish diabetes prevention study (DPS): lifestyle intervention and 3-year results on diet and physical activity. Diabetes Care.

[48] Duijzer G, Haveman-Nies A, Jansen SC, ter Beek J, van Bruggen R, Willink MGJ (2017). Effect and maintenance of the SLIMMER diabetes prevention lifestyle intervention in Dutch primary healthcare: a randomised controlled trial. Nutr Diabetes.

[49] Lu J, ManLam S, Wan Q, Shi L, Huo Y, Chen L (2019). High-coverage targeted lipidomics reveals novel serum lipid predictors and lipid pathway dysregulation antecedent to type 2 diabetes onset in normoglycemic Chinese adults. Diabetes Care.

[50] Kontush A (2015). HDL particle number and size as predictors of cardiovascular disease. Front Pharmacol.

[51] Frazier-Wood AC, Manichaikul A, Aslibekyan S, Borecki IB, Goff DC, Hopkins PN (2013). Genetic variants associated with VLDL, LDL and HDL particle size differ with race/ethnicity. Hum Genet.

[52] Busher JT (1990). Serum albumin and globulin. Clinical methods: the history, physical, and laboratory examinations.

[53] Jun JE, Lee SE, Lee YB, Jee JH, Bae JC, Jin SM (2017). Increase in serum albumin concentration is associated with prediabetes development and progression to overt diabetes independently of metabolic syndrome. PLoS ONE.

[54] Kunutsor SK, Khan H, Laukkanen JA (2015). Serum albumin concentration and incident type 2 diabetes risk: new findings from a population-based cohort study. Diabetologia.

[55] Barnett AG (2004). Regression to the mean: what it is and how to deal with it. Int J Epidemiol.

