# Supplementary material for: Clinical and metabolomic predictors of regression to normoglycemia in a population at intermediate cardiometabolic risk
Source: Cardiovasc Diabetol. 2021 Feb 27;20:56. doi: 10.1186/s12933-021-01246-1 (PMC7916268; doi:10.1186/s12933-021-01246-1)
Supplement: Supplementary file 1 — Additional file 1: Table S1. Metabolites associated with regression to normoglycemia compared with progression to type 2 diabetes. [file 12933_2021_1246_MOESM1_ESM.docx]

| **Table S1.** Metabolites associated with regression to normoglycemia compared with progression to type 2 diabetes | | | |
| --- | --- | --- | --- |
|  | **HR** | **CI 95%** | ***p value*** |
| Albumin | 0.732 | 0.59- 0.90 | 0.003 |
| Cholesterol esters in very large HDL | 1.32 | 1.09 – 1.61 | 0.004 |
| Total lipids in very large HDL | 1.31 | 1.07 – 1.59 | 0.007 |
| Concentration of large HDL particles | 1.30 | 1.07 – 1.59 | 0.007 |
| Total cholesterol in very large HDL | 1.29 | 1.06 – 1.57 | 0.009 |
| Cholesterol esters in chylomicrons and extremely large VLDL | 1.25 | 1.04 – 1.51 | 0.017 |
| Triglycerides in large VLDL | 0.78 | 0.63 – 0.96 | 0.023 |
| Phospholipids in medium LDL | 0.78 | 0.63 – 0.96 | 0.023 |
| Free cholesterol in small HDL | 0.82 | 0.67 – 1.00 | 0.024 |
| Free cholesterol in small LDL | 0.79 | 0.64-0.97 | 0.024 |
| Triglycerides in large HDL | 1.24 | 1.02 - 1.51 | 0.029 |
| Total cholesterol in chylomicrons and extremely large VLDL | 1.22 | 1.01 -1.47 | 0.030 |
| Phospholipids in large LDL | 0.79 | 0.64 – 0.98 | 0.033 |
| Phospholipids in very large HDL | 1.23 | 1.01 – 1.51 | 0.036 |
| Phospholipids in very large VLDL | 0.80 | 0.65 - 0.98 | 0.038 |
| Phospholipids in IDL | 0.80 | 0.64 - 0.99 | 0.040 |
| Total cholesterol in large VLDL | 1.24 | 1.00 – 1.54 | 0.045 |
| Citrate | 1.23 | 1.00-1.52 | 0.049 |
| HR: Hazard Ratio and their CI 95%. Models were adjusted by baseline variables: age, sex, body mass index, and fasting glucose | | | |
